# Fluid resuscitation should respect the endothelial glycocalyx layer

**DOI:** 10.1186/s13054-014-0707-6

**Published:** 2014-12-23

**Authors:** Bertrand Guidet, Hafid Ait-Oufella

**Affiliations:** Assistance Publique - Hôpitaux de Paris, Hôpital Saint-Antoine, Service de Réanimation Médicale, 184 rue du Faubourg Saint Antoine, Paris, F-75012 France; Sorbonne Universités, UPMC Univ Paris 06, UMR_S 1136, Institut Pierre Louis d’Epidémiologie et de Santé Publique, 47-83 Boulevard de l’hôpital, Paris, 75013 France; INSERM, UMR_S 1136, Institut Pierre Louis d’Epidémiologie et de Santé Publique, 47-83 Boulevard de l’hôpital, Paris, 75013 France; Inserm U970 PAris Research Cardiovascular Center (PARCC), 56 rue Leblanc, Paris, 75015 France

## Abstract

Endothelial glycocalyx degradation induced by fluid overload adds to the concern of a detrimental effect of uncontrolled fluid resuscitation and the risk of unnecessary fluid infusion. As a consequence, the use of new tools for monitoring response to fluids appears promising. From that perspective, the monitoring of plasma concentration of glycocalyx degradation markers could be useful.

Fluid resuscitation is common practice for patients with hypovolemia and is recommended as first-line treatment for septic shock. However, accumulating evidence suggests that uncontrolled fluid loading may be detrimental. In that respect, the study by Chappell and colleagues [1] in the previous issue of *Critical Care* adds an important piece of information to the debate over the need for careful monitoring of patients in order to avoid unnecessary fluids.

During peri-operative management, Chappell and colleagues prospectively explored the consequences of acute volume loading on endothelial functions and hypothesized that high-volume infusion could alter endothelial glycocalyx. The glycocalyx consists of a variety of endothelial membrane-bound molecules, including glycoproteins and proteoglycans that form a negatively charged barrier to circulating cells and macromolecules [2]. Numerous animal studies have demonstrated that destruction of the glycocalyx (using enzymatic approaches, for example) leads to increased capillary permeability [3]. *In vivo*, degradation of the glycocalyx with heparinase favors tight contacts between circulating leukocytes and the endothelium through denudation of the protective barrier that normally prevents this effect [4]. In addition, the glycocalyx participates in the coagulation process as heparan sulfate and dermatan sulfate, two glycocalyx glycosaminoglycans, potentiate the activity of two anticoagulant enzymes: antithrombin III (by a factor of 100) and heparin cofactor II, respectively (reviewed in [5]).

Chappell and colleagues reported that volume loading (20 mL/kg of hydroxyethyl starch (HES) 130/0.4) in elective surgery induced a release of atrial natriuretic peptide (ANP) associated with an increase of serum glycocalyx components (syndecan-1 and hyaluronan) but that there was no effect of acute normovolemic hemodilution. The augmentation of these plasma components suggests a degradation of endothelial glycocalyx that could promote vascular leakage, leukocyte adhesion, and procoagulant status. However, this is a speculative and physiopathological conclusion, and the authors did not provide any direct evidence of glycocalyx functional alteration. The twofold increase of serum syndecan-1 concentration after volume overload is statistically significant but was very far from the 10-fold increase reported during septic shock [6] and the 40-fold increase after aortic surgery [7].

The mechanisms of glycocalyx shedding have not been directly investigated, but the authors proposed that ANP could be implicated. This hypothesis is based on a previous experimental study by the same group [8] and the observation of a simultaneous plasma increase of ANP and glycocalyx components during volume overload. Other mechanisms of shedding have been proposed [5], such as the activation of circulating enzymes (heparinase, neuraminidase, and pronase), the release of pro-inflammatory cytokines (tumor necrosis factor-alpha), or the direct effect of increased shear stress on the arterial endoluminal wall. Finally, reactive oxygen species could also induce glycosaminoglycans release. As albumin has anti-oxidant properties [9], one can speculate that volume resuscitation using human albumin could be less harmful than HES. A recent trial suggests that albumin could reduce mortality in the most severe cases of septic shock [10]. The mechanisms of the potential beneficial effect of albumin remain unclear. An interaction of albumin with the glycocalyx leading to a reduction of the capillary leak is a seductive hypothesis but should be further investigated [11].

This translational study gives interesting insights into the deleterious effects of fluid infusion in critically ill patients. Indeed, accumulative clinical studies pointed out that positive fluid balance and weight gains are associated with a worse prognosis [12] but the mechanisms of this deleterious effect remain unknown. Fluid accumulation within the tissue could alter the diffusion of oxygen, promoting hypoxic cellular injury. Fluid resuscitation is recommended as a first-line treatment, particularly during the first hour [13], but the volume and type of fluid that should be used remain controversial. The choice of fluid should be integrated in a dynamic process (rescue, optimization, stabilization, and de-escalation) [14]. Recent trials have added to the confusion regarding the usefulness of an algorithm guiding hemodynamic management of shock [15] and the mean arterial pressure that should be targeted [16]. Three criteria should be present: evidence of fluid responsiveness such as pulse pressure variation; presence of signs of tissue hypoperfusion; and absence of fluid overload. Stopping rules are either no fluid responsiveness or absence of signs of tissue hypoperfusion or presence of fluid overload. We need to set clinically relevant goals. From that perspective, an integrative approach looking at simple indices of tissue perfusion and microcirculation could be useful at the bedside [17,18], although so far no interventional study has proven that such a strategy is able to improve the outcome. The protection or restoration of glycocalyx might be considered an important goal [19]. The place of glycocalyx degradation components remains to be assessed, but they could represent a surrogate marker for physicians to identify vascular endothelial injury and ultimately to limit fluid overload. A respectful strategy for the glycocalyx may decrease the capillary leak and improve tissue oxygenation just by giving the right amount of fluids and maybe by choosing fluids with no impact on glycocalyx degradation.

## Abbreviations

ANP: Atrial natriuretic peptide
HES: Hydroxyethyl starch
